# A comparative study of ultrasound cycloplasty and endoscopic cyclophotocoagulation in the treatment of secondary glaucoma

**DOI:** 10.1038/s41598-023-50157-6

**Published:** 2023-12-27

**Authors:** Wang Ruixue, Ding Wenjun, Jiang Le, Fan Fangfang, Li Ning, Chen Xiaoya, Li Suyan

**Affiliations:** 1 The Affiliated Xuzhou Municipal Hospital of Xuzhou Medical University, Xuzhou, Jiangsu China; 2 Department of Ophthalmology, Xuzhou First People’s Hospital, Xuzhou, Jiangsu China; 3 Department of Ophthalmology, The First Affiliated Hospital of Anhui Medical University, Hefei, Anhui China

**Keywords:** Diseases, Medical research

## Abstract

To compare the clinical efficacy of ultrasound cycloplasty (UCP) and endoscopic cyclophotocoagulation (ECP) in the treatment of secondary glaucoma. In a 12-month prospective single-center study, 22 patients with secondary glaucoma were treated by high-intensity focused ultrasound (HIFU), and 23 patients with secondary glaucoma were treated by a semiconductor laser. At the final follow-up, the two groups’ surgical outcomes were compared. A complete success was defined as an intraocular pressure (IOP) reduction of at least 20% from baseline and an IOP of > 5 mmHg and ≦ 21 mmHg, while a qualified success was defined as an IOP reduction of at least 20% from baseline and an IOP of > 5 mmHg. The secondary outcome was the average IOP, number of drugs, and complications at each follow-up compared with the baseline. The average preoperative IOPs in the UCP and ECP groups were 36.4 ± 9.5 mmHg (n = 2.3 drops, n = 0.2 tablets) and 34.5 ± 11.7 mmHg (n = 2.0 drops, n = 0.3 tablets), respectively. In the last follow-up, the success rate of UCP was 54% (with a decrease of 32%) and that of ECP was 65% (with a decrease of 35%), and the P-value between the two groups was > 0.05. However, there was a difference in the average IOP between these two groups 1 day and 1 week after the operation, and the IOP reduction efficiency in the ECP group was better. However, the amount of drug used after these two surgeries was significantly reduced. There were fewer postoperative complications in the UCP group (18 cases) than in the ECP group (35 cases). Both UCP and ECP can effectively reduce IOP in secondary glaucoma, and ECP has a better effect at the early stages. However, UCP has higher safety and tolerance for patients.

## Introduction

Secondary glaucoma is an eye syndrome that is caused by some eye or systemic diseases and some unreasonable drugs that interfere with the normal aqueous humor circulation, leading to an increase in intraocular pressure (IOP). Compared with primary glaucoma, its condition is more severe and complicated. Early detection and treatment of primary disease is very important^1^. Common primary diseases include inflammation, trauma, vascular diseases, related syndromes, drugs, and so on. Long-term and stable control of IOP is the key measure to treat secondary glaucoma^2,3^. At present, the treatment methods include local or systemic medication to reduce IOP, iris laser, filtration surgery to increase aqueous humor drainage, and ciliary body destruction surgery to reduce aqueous humor^4^.

There are various mechanisms and targets in clinical medicine and surgery. Both ultrasound cycloplasty (UCP) and endoscopic cyclophotocoagulation (ECP) discussed in this study belong to ciliary destruction surgery and have specific targeting^5^. Both of them produce thermal effects by absorbing external energy, which leads to the coagulation necrosis of target tissue, thus effectively controlling the generation of aqueous humor^6,7^. However, there are differences in the destruction mode between the two operations. UCP forms directional and gentle local thermal coagulation of the ciliary process through high-intensity focused ultrasound (HIFU). Because the absorption degree of ultrasonic waves has nothing to do with pigment, the energy released into the tissue can be accurately controlled^8^. ECP relies on the pigment content and absorption coefficient of the ciliary epithelium, using semiconductor lasers to accurately destroy a certain number of ciliary processes. Its advantage is that the operator can directly look at the target tissue with the assistance of an endoscope, avoiding the blindness of surgery^9^. Compared with traditional transscleral diode laser cyclophotocoagulation, it not only expands the visual field of the operator but also reduces the damage to the sclera and the incidence of related complications^10,11^.

In recent years, the efficacy and safety of these two surgical treatments for glaucoma have been gradually recognized in clinical practice^12,13^. However, currently, there has been no research report on the comparison between UCP and ECP for secondary glaucoma. The increase in IOP after simple cataract surgery or combined artificial lens implantation is one of the most important types of secondary glaucoma. The sample of this study is aphakia or pseudophakic patients. We hope to compare the differences in postoperative IOP, dosage, and complications between the two operations through this study.

## Materials and methods

### Patients

This is a prospective study. In the UCP group, 22 eyes of 22 patients were treated from November 2017 to September 2022. In the ECP group, 23 eyes of 23 patients were treated from January 2019 to November 2022. The age range of patients in the UCP and ECP groups in this study is between 25 and 80 years old and 35 and 88 years old, respectively. This research was authorized by the appropriate institutional review board (Ethics Review Committee of Xuzhou First People's Hospital) and carried out in compliance with the regulations set forth in the Declaration of Helsinki and ISO 14,155 standards. Written informed consent was obtained from all enrolled patients.

### Inclusion and exclusion criteria

The thickness and curvature of the lens cannot allow the endoscopic probe to freely access and aim, and ECP is also difficult to avoid, leading to the occurrence and development of cataracts. Therefore, in this study, we selected patients who had undergone cataract surgery.

The inclusion criteria were as follows: (1) patients diagnosed with secondary glaucoma, (2) hypotensive medication being insufficient to control the IOP, (3) IOP greater than or equal to 20 mmHg, (4) age greater than 18 years old and less than 90 years old, (5) no intraocular surgery or laser treatment 90 days before surgery, and (6) patients who signed the informed consent and who were able to complete all postoperative follow-up visits.

The exclusion criteria were as follows: (1) eye infection in any eye in the 2 weeks before treatment; (2) pregnant or lactating women; and (3) any medical or treatment history or systemic disease that may affect the evaluation of the treatment efficacy.

### Preoperative examination

Routine eye examination was performed before the operation, including uncorrected visual acuity, photography of the anterior segment, gonioscopy, fundus photography, and IOP measurement (Goldman tonometer). Furthermore, an ultrasound biomicroscope (UBM) should be performed before UCP, in addition to measuring the axial length and white-to-white distance.

### Treatment procedure

Both anesthesia and treatment were performed by the same experienced ophthalmologist. Retrobulbar anesthesia was applied to all patients. Antibiotic eye drops and steroid eye drops were added one month after surgery. The initial frequency was 4 times a day, and then the steroid eye drops gradually decreased.

UCP: The study used an EyeOP1 device imported from France, which has been described in detail previously^14,15^. According to the diameter, the therapeutic probe can be divided into three types: 11 mm, 12 mm, and 13 mm, which can be selected reasonably according to the patient’s eye condition. The exposure time was 8 s, and the number of sectors was 8. (1) The patient was supine on the surgical bed. After starting the instrument, the positioning ring was taken out, adsorffbed on the ocular surface, and centered. (2) Negative pressure detection confirmed that there was no suction loss. The probe was placed in the positioning ring and filled with normal saline, and the treatment was started by stepping on the foot. (3) After the 6-sector treatment is completed, the probe is rotated to perform the remaining-sector treatment. (4) After the treatment, the probe and the positioning ring were taken out. The patients stayed in the hospital for observation for 2 h.

ECP: The URAM-E4 laser endoscope system imported from the United States was used in this operation. The number of ciliary bodies in all patients who received photocoagulation was 30. The specific steps were as follows: (1) 30 min before surgery, compound topicamide fully dilated pupil. (2) A main corneal incision of about 3.2 mm was made at 11 o’clock, and the laser probe entered the eye through the incision with the assistance of a viscoelastic agent. (3) It was observed and illuminated with the help of an endoscope system, with which the ciliary process was focused, and photocoagulation treatment was started in each quadrant^16^. The parameters were adjusted according to the photocoagulation reaction during the treatment. The best reaction is that the ciliary body turns white, collapses, and shrinks. If the ciliary body tissue is weak, the laser power is small. If the ciliary body tissue bursts, the energy is too strong. Patients can only be discharged after 1 to 3 days of hospitalization observation.

### Postoperative follow-up

Follow-up visits were scheduled on day 1, week 1, month 1, month 3, month 6, and month 12 after treatment. Eye examinations were performed at every visit, such as uncorrected visual acuity, photography of the anterior segment, IOP measurement (Goldman tonometer), and complication evaluation.

### Outcome measures

The main outcome was that the operation was successful at the last follow-up, and the secondary outcome was the average IOP, drug use, and complications compared with the baseline at each follow-up^12^.

Complete surgical success criteria: IOP decreased by ≥ 20% from the baseline, 5 mmHg < IOP ≤ 21 mmHg.

Qualified surgical success criteria: IOP decreased by ≥ 20% from the baseline, IOP > 5 mmHg.

### Statistical analysis

The data were analyzed using SPSS 23.0 statistical software (IBM, USA). Descriptive statistics were used to report the demographic and ocular baseline characteristics. The student’s *t* test, χ^2^ test, and Fisher exact test were used for demographic analysis, and the student’s *t* test was used to compare the differences between groups of continuous variables. A P-value of < 0.05 was considered statistically significant.

## Results

### Patient characteristics

In this study, both groups of patients successfully completed the operation. Patient details are shown in Table 1.

**Table 1:** Patients characteristics.

	UCP	ECP	P
Patients	22	23	
Age, mean ± SD (range), year	60.3 ± 14.9 (25–83)	65.7 ± 12.9 (31–82)	0.208^a^
Sex (male/female)	12/10	8/15	0.182^b^
Type of glaucoma			0.338^c^
SACG	9	11	
SOAG	7	9	
NG	6	2	
Ocular hypertension	0	1	
Axial length, mm	23.7 ± 1.9	23.3 ± 0.8	0.341^a^
WTW, mm	11.6 ± 0.7	11.6 ± 0.7	0.877^a^
Lens status			0.608^c^
Pseudophakic	20	22	
Aphakic	2	1	
IOP baseline, mean ± SD	36.4 ± 9.5	34.5 ± 11.7	0.557^a^
Preoperative hypotensive medications, mean ± SD			
Drops	2.3 ± 0.6	2.0 ± 0.6	0.146^a^
Tablets	0.2 ± 0.4	0.3 ± 0.5	0.350^a^
Visual acuity, LogMar			0.767^c^
Visual acuity	8	11	
Count fingers	3	1	
Hand motion	7	7	
Light perception	1	2	
No light perception	3	2	

*SD* standard deviation, *SACG* secondary open-angle glaucoma, *SOAG* secondary angle-closure glaucoma, *NG* neovascular glaucoma, *WTW* white to white, *IOP* intraocular pressure.

^a^Student’s *t*-test, ^b^χ^2^ test, ^c^Fisher test.

### Intraocular pressure

Each follow-up of IOP in all patients is shown in Table 2. In the UCP and ECP groups, the average IOP at 1 day, 1 week, 1 month, 3 months, 6 months, and 12 months after surgery was statistically different from the baseline IOP (P < 0.05). At 1-day and 1-week follow-up after surgery, there were differences between the two groups. The IOP in the ECP group was lower, and the IOP decreased more than that in the UCP group. However, there was no significant difference in IOP control between the two groups during follow-up.

**Table 2:** Intraocular pressure at baseline and during follow-up in the patients.

Catalogue	UCP			ECP			P value^a^
	Mean ± SD IOP (no patients)	Relative IOP reduction (%)	P value compared with the baseline^a^	Mean ± SD, IOP (no patients)	Relative IOP reduction (%)	P value compared with the baseline	
Baseline	36.4 ± 9.5 (22)	NA	NA	34.5 ± 11.7 (23)	NA	NA	0.375
Day 1	25.5 ± 9.7 (22)	27	0.001	15.8 ± 4.6 (23)	47	0.000	0.000
Day 7	20.9 ± 6.0 (21)	40	0.000	16.4 ± 5.7 (23)	48	0.000	0.017
Month 1	18.8 ± 8.6 (22)	46	0.000	16.3 ± 6.4 (20)	46	0.000	0.311
Month 3	20.8 ± 9.4 (19)	42	0.000	18.9 ± 5.3 (19)	38	0.000	0.450
Month 6	19.9 ± 7.7 (18)	41	0.000	20.4 ± 8.8 (18)	38	0.000	0.846
Month 12	22.3 ± 9.6 (16)	32	0.000	19.4 ± 6.6 (19)	35	0.000	0.337

*NA* not applicable, *IOP* intraocular pressure, *SD* standard deviation.

^a^Student’s *t*-test.

**Figure 1:**
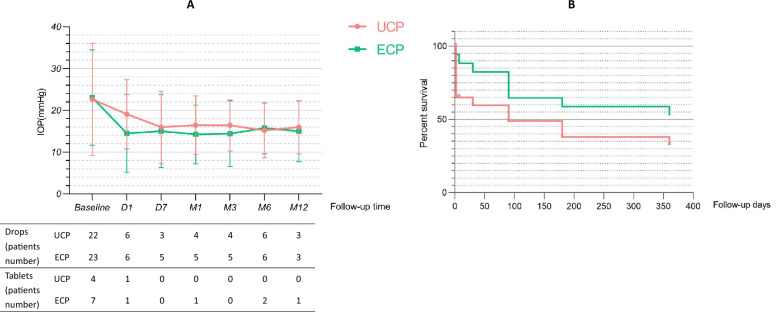
Baseline and postoperative IOP at each follow-up visit, with the corresponding number of hypotensive drops and tablets used (**A**). The Kaplan-Maier curves analyzed (**B**).

**Figure 2:**
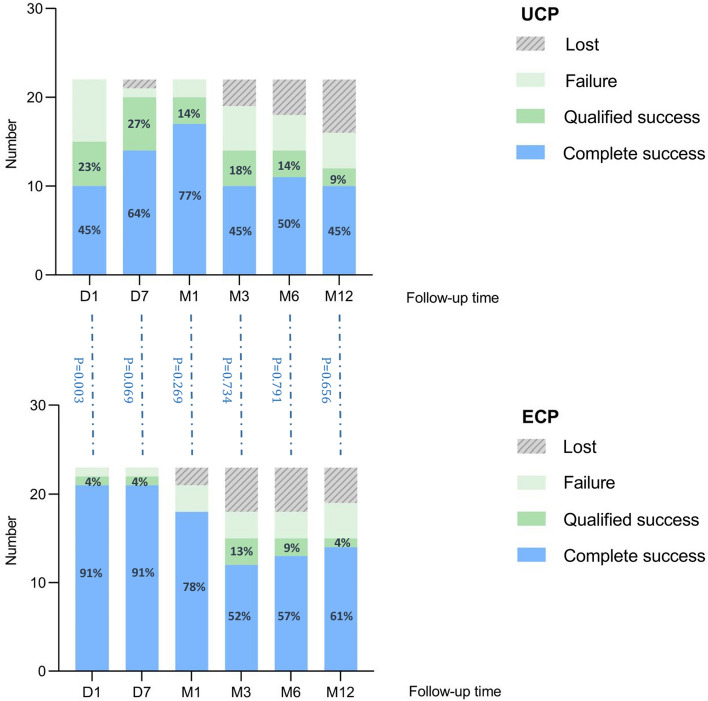
Success rates and corresponding operative result number at each follow-up visit in the study.

Figure 1 shows the trend of the average IOP at each follow-up, the number of patients using hypotensive medication drugs, and the Kaplan-Maier curve of cumulative success rates. There was no difference in the number of hypotensive medication drops and tablets between the two groups before the operation, and the average number of drugs used 1 year after the operation was significantly reduced. The complete success rate and qualified success rate of the operation are shown in Fig. 2.

### Complications

Complications occurred in both groups, including scleral marks and eye pain during and after surgery. There were more patients with scleral marks in the UCP group, and ECP did not need to pass through the sclera, so no such complications occurred.

All the related complications of the two groups during the follow-up period are shown in Table 3, and the patients with treatment failure and poor results are shown in Table 4.

**Table 3:** Intraoperative and postoperative complications.

Complications	Ocular complications	UCP	ECP
Intraoperative	Scleral marks	20	0
	Ocular pain	1	2
Postoperative	Scleral marks	6	0
	Ocular pain	4	5
	Transient visual impairment	1	2
	Loss of vision > 2 lines at last follow-up	1	0
	Conjunctival hyperemia	3	11
	Corneal edema	2	3
	Hyphema	1	0
	Aqueous flare	1	14
	Hypotony	2	1

**Table 4:** Cases of surgical failure or insufficient effect.

Patient	Gender	Age	Glaucoma type	Baseline IOP	No. baseline drugs	Last IOP (months)	No. last drugs	Outcome
UCP								
1	Female	37	NV	40	3	21 (6)	0	Tube
2	Male	59	NV	48	3	40 (12)	0	Hyphema
3	Male	67	SACG	55	3	42 (3)	0	Tube
4	Female	83	SOAG	30	3	42 (12)	3	Trab
ECP								
1	Male	70	SACG	21	3	40 (12)	3	UCP
2	Female	81	SACG	26	3	37 (1)	2	Tube
3	Female	66	SACG	58	3	26 (12)	0	UCP
4	Female	69	NV	61	2	42 (6)	2	Tube

Overall, the UCP group had fewer postoperative complications than the ECP group, especially when the inflammatory reaction was lighter. There were 18 cases of postoperative complications in the UCP group and 35 cases in the ECP group.

### Ciliary body

**Figure 3:**
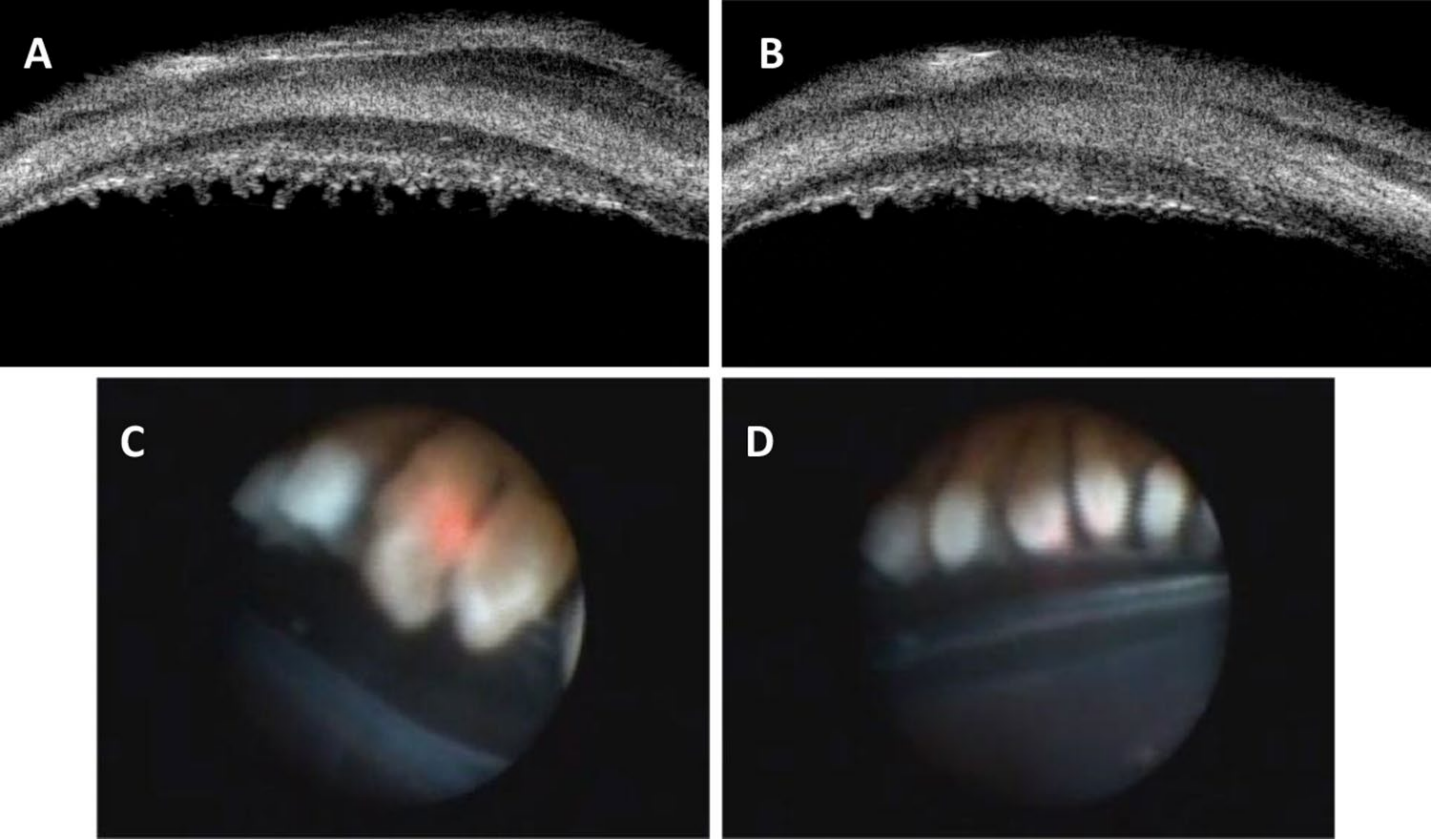
UBM examinations the ciliary morphology in the UCP group (**A**), preoperative (**B**), postoperative. Note that the areas of treatment are locatedat the junction between the sclera and the base of the ciliary body. View during endoscopic cyclophotocoagulation (**C**), the right two ciliary processes were preoperative (**D**), shrunken and treated processes. Note that there is less iridociliary contact compared with natural state because of the sulcus being inflated with viscoelastic.

The UBM images before and after UCP treatment are shown in Fig. 3A,B, and one significant lesion area induced by HIFU can be seen in the ciliary body of the patient. An endoscopic photograph of ECP is shown in Fig. 3C,D. The ciliary body was large before the operation, and the ciliary process shrank and turned white after being treated with a semiconductor laser.

## Discussion

Secondary glaucoma is a relatively complex group of glaucoma that usually has a serious primary disease, and the choice of treatment is more complicated. Traditional medicine, lasers, and filtering surgery sometimes cannot control the IOP stably. In the past, cyclocryotherapy was used, but the low-temperature effect caused significant damage to the eye tissue, the patient’s pain was obvious, and serious complications often occurred^5,17^. Since the 1990s, ciliary body photocoagulation using laser energy combined with endoscopy has been used in clinical practice, and more ideal therapeutic effects have been obtained. It not only effectively reduces IOP but also reduces the occurrence of complications^18,19^. With the progress of medical technology, HIFU technology has developed rapidly, and UCP has become a widely used method to treat glaucoma in recent years^20^. This study aims to compare the efficacy and safety of the latter two kinds of ciliary body destructive surgery in the treatment of secondary glaucoma.

During the 1-year observation, we found that both can effectively reduce IOP. The average baseline IOP of the two groups was similar, and the average IOP of each follow-up was statistically different from the baseline (P < 0.05). For UCP and ECP groups, in the final follow-up, the surgeries for most patients were successful; the success rates were 54% and 65%, and the complete success rates were 45% and 61%, respectively. However, there were differences in IOP decreasing effects between the two groups, as shown in Table 2 and Fig. 2. One day after the operation, the average IOP in the UCP group did not drop to the normal range. Although it was 27% lower than the baseline, it was much lower than that of the ECP group, and the decline of the ECP group was as high as 47%. One week after the operation, the average IOP in the UCP group decreased to 20.9 ± 6.0 mmHg and achieved ideal results. The average IOP in the ECP group was 16.4 ± 5.7 mmHg. There were still differences between the two groups in this follow-up, but there was no significant difference in the success rate of surgery. This result shows that UCP’s early IOP lowering effect is not as good as ECP’s to some extent, which may be related to the milder effect of HIFU on the ciliary body than that of the semiconductor laser, or it may be due to insufficient ultrasound dose and weakened thermal coagulation on tissues, which makes the IOP lowering effect not appear immediately^15,21^. At the same time, UCP has a dual mechanism, which can not only reduce the generation of aqueous humor but also open the local choroidal scleral channel, which may not be fully functional on the first day after the operation^22,23^. During the 1-week follow-up, the average IOP in the UCP group dropped to a normal level. In the subsequent follow-up, UCP and ECP achieved satisfactory results in lowering IOP and the success rate of surgery. In the end, 12 patients in the UCP group and 15 patients in the ECP group were successfully operated on, and the IOP decreased by 32% and 35%, respectively. There was no difference in IOP control between the two groups. However, the cumulative success rates of the two are significantly different, as shown in Fig. 1B. In our survival analysis of the two groups, the Kaplan-Maier curve showed that the cumulative success rate of the ECP group was consistently higher than that of the UCP group, and the number of successful patients was higher. This indicates that ECP may be more efficient at reducing IOP to a certain extent.

In our study, all patients stopped using IOP-lowering drugs after the operation and used drugs again only when the IOP was found to be more than > 21 mmHg at each follow-up. Most of these patients can maintain normal IOP through re-medication^24^. This is because there are complications and side effects of hypotensive medication drugs, especially tablets (acetazolamide), which may cause paresthesia, metabolic acidosis, electrolyte changes, and even serious hematological diseases^25–27^. Therefore, we believe that maintaining the same drugs as before the operation when the IOP value is within the normal range not only makes the patient bear a heavy treatment burden but also increases the potential risk^28^. We counted the numbers of patients who used hypotensive medication drops and tablets at each follow-up before and after surgery, and obtained the average IOP lines of those patients, as shown in Fig. 1 (A). The number of patients using hypotensive medication drops and tablets on the first day after surgery was significantly reduced in both groups. At subsequent follow-up, the numbers of patients using hypotensive medication drops remained stable and less than 6, and no patient in the UCP group took tablets after 1 week.

As far as surgical safety is concerned, most adverse events are mild and transient. UCP has the advantage of outstanding safety. The two operations have different ways of controlling the dose. UCP supports the selective application of the ciliary process through a computer, which is quantitative and has strict temperature control^29^. Although ECP can set the laser energy, the stay time and range of the probe in the ciliary body are artificially operated, and there is a certain degree of error. Scleral marks are a unique complication of UCP, which are caused by thinning the sclera when HIFU passes through it, corresponding to the treatment sector, and there is no inflammatory reaction^30,31^. All scleral marks disappeared within six months after the operation, and most patients disappeared within three months after the operation. One of the surgical purposes of UCP and ECP is to relieve patients’ pain and anxiety. During the operation, one patient in both groups felt mild pain, possibly due to the different tolerances of patients to anesthetics. After the operation, patients in the UCP group felt less pain than those in the ECP group. Some patients have been relieved after taking medicine, and some patients need to have other IOP reduction surgeries. Patients in both groups suffered from transient visual impairment, which disappeared spontaneously within 1 week^32,33^. Postoperative corneal edema is the main reason. Only one patient in the UCP group was not recovered from vision loss at the last follow-up. According to our observations, this patient had a posterior cataract during the follow-up, and the posterior capsule opacity gradually increased. However, ECP has a higher incidence of inflammatory reactions and more serious symptoms than UCP. Both surgeries stimulate the conjunctival tissue and cause vasodilation and congestion. There were 11 cases of conjunctival hyperemia after the operation in the ECP group and only 3 cases in the UCP group. At the same time, patients in the ECP group have more aqueous flare because the probe needs to enter the eye through a corneal incision, which will cause some physical and chemical damage to corneal tissue, especially endothelial cells, during the operation. At the same time, inflammatory cytokines and protein fragments released by ciliary body necrosis can trigger inflammatory reactions. There were also two cases of aqueous flare in the UCP group, which may be secondary to the direct injury of ciliary pigment epithelial cells, and the pigment epithelial layer is a key component of the blood-aqueous barrier^34,35^. Despite the continuous development of ophthalmic surgery technology, including minimally invasive surgery such as UCP, a certain degree of intraocular inflammation is still inevitable after surgery.

A total number of eight patients had serious results, with four patients in each group. A patient with hyphema occurred after UCP. This patient had a history of diabetes for 9 years, had poor blood-aqueous barrier function, and was prone to leakage and bleeding when IOP dropped. The patient was instructed to take a semi-recumbent position and, at the same time, use hormone eye drops and oral hemostatic drugs. After one month, the bleeding subsided. In the UCP group, one patient’s IOP fluctuated after the operation, and the IOP was at a critical value at six months’ review. At eight months’ time, valve tube implantation was performed because the IOP was difficult to control. The patient is young, and the function of ciliary epithelial cells is easily reactivated. One patient’s IOP was always higher than normal after the operation, and three kinds of hypotensive medication drugs were used before and after the operation, but the IOP was still as high as 42 mmHg three months after the operation, and valve tube implantation was performed 3 days later. Another patient had a trabeculectomy after the last follow-up. The postoperative IOP of the patient was well controlled and then suddenly increased at the last follow-up. This is probably because of the insufficient destruction of the ciliary body or excessive secretion of aqueous humor due to the compensation of the residual ciliary body. There were also patients with poor effects in the ECP group, and two patients tried UCP treatment after ECP for 1 year. Among them, the IOP of one patient was normal within one month after operation, but its value was always > 30 mmHg in the subsequent review. This patient’s baseline IOP was high, and the number of damaged ciliary bodies may make it difficult to prevent the IOP from rising. Another patient had only a slight increase in IOP at the last follow-up. One patient’s IOP began to increase 1 week after the operation. Although we kept hypotensive medication drugs consistent with those before the operation, it was still difficult to control. The patient underwent valve tube implantation 2 months later. The last patient’s IOP began to rise 3 months after the operation, and after seven months, this patient was followed up by telephone to perform valve tube implantation in the other hospital.

In order to feel the degree of damage to the ciliary body more intuitively, we used UBM to scan the ciliary body in the treatment area before and after UCP and observe its shape and quantity. The ciliary epithelial tissue after HIFU treatment was thermally coagulated, and some tissues were lost, as shown in Fig. 3A,B^33^. In the process of ECP implementation, we also photographed the ciliary body before and after the laser, and some tissues turned white quickly and became smaller after laser implementation, as shown in Fig. 3C,D^5,36^.

This study's defect is mainly caused by the limited clinical applications of these two surgeries. During the follow-up period, we flexibly adjusted the treatment plan according to the patient’s IOP and the overall situation of the eye, solved the patient’s current condition in time, and implemented other surgical rescue treatments when necessary. This led to an increase in the number of patients who dropped out of the study group due to illness, in addition to the lost patients. However, this research method is more ethical and humane. In addition, no patients were treated twice, and we cannot be sure about the safety of patients who have been treated many times. At the same time, the number of patients in the two groups is small, and larger multicenter research and long-term efficacy evaluations are needed in the future.

In summary, UCP and ECP have a high success rate in the treatment of secondary glaucoma, and they can not only reduce the IOP level but also reduce the use of hypotensive medication drugs. The difference between the two is that ECP has a more significant effect on lowering IOP in the early stage, and the complete success rate is higher, while UCP has better safety and comfort, which not only shortens the treatment time but also has fewer postoperative complications. Meanwhile UCP does not require a microscopic operation, and doctors can observe the display screen for surgery, which greatly shortens the learning curve^5^. Both these two surgeries are valuable tools for the treatment of secondary glaucoma. They are effective and well tolerated, making them effective supplements to glaucoma surgery.

## Data Availability

The datasets generated during and/or analysed during the current study are available from the corresponding author on reasonable request.
